# A Possible Role for Nerve Growth Factor and Its Receptors in Human Sperm Pathology

**DOI:** 10.3390/biomedicines11123345

**Published:** 2023-12-18

**Authors:** Anna Maria Stabile, Alessandra Pistilli, Elena Moretti, Desirée Bartolini, Mariangela Ruggirello, Mario Rende, Cesare Castellini, Simona Mattioli, Rosetta Ponchia, Sergio Antonio Tripodi, Giulia Collodel

**Affiliations:** 1 Department of Medicine and Surgery, Section of Human, Clinical and Forensic Anatomy, University of Perugia, 06132 Perugia, Italy; anna.stabile@unipg.it (A.M.S.); alessandra.pistilli@unipg.it (A.P.); mariangela11@live.it (M.R.); mario.rende@unipg.it (M.R.); 2 Department of Molecular and Developmental Medicine, University of Siena, 53100 Siena, Italy; giulia.collodel@unisi.it; 3 Department of Pharmaceutical Sciences, Section of Biochemistry, University of Perugia, 06132 Perugia, Italy; desirée.bartolini@unipg.it; 4 Department of Agricultural, Environmental and Food Science, University of Perugia, 06100 Perugia, Italy; cesare.castellini@unipg.it (C.C.); simona.mattioli@unipg.it (S.M.); 5 Unit of Medically Assisted Reproduction, Siena University Hospital, 53100 Siena, Italy; ponchia2@student.unisi.it; 6 Department of Pathology Unit, Azienda Ospedaliera Universitaria Senese, 53100 Siena, Italy; tripodis@unisi.it

**Keywords:** human sperm, neurotrophins, varicocele, TrKA and p75^NTR^, urogenital infections

## Abstract

Nerve growth factor (NGF) signalling affects spermatogenesis and mature sperm traits. In this paper, we aimed to evaluate the distribution and the role of NGF and its receptors (p75^NTR^ and TrKA) on the reproductive apparatus (testis and epididymis) and sperm of fertile men (F) and men with different pathologies, namely varicocele (V) and urogenital infections (UGIs). We collected semen samples from 21 individuals (31–40 years old) subdivided as follows: V (*n* = 7), UGIs (*n* = 7), and F (*n* = 7). We submitted the semen samples to bacteriological analysis, leucocyte identification, and analysis of sperm parameters (concentration, motility, morphology, and viability). We determined the seminal plasma levels of NGF, interleukin 1β (IL-1β), and F_2_-isoprostanes (F_2_-IsoPs), and the gene and protein expression of NGF receptors on sperm. We also used immunofluorescence to examine NGF receptors on ejaculated sperm, testis, and epididymis. As expected, fertile men showed better sperm parameters as well as lower levels of NGF, F_2-_IsoPs, and IL-1β compared with men with infertility. Notably, in normal sperm, p75^NTR^ and TrKA were localised throughout the entire tail. TrKA was also found in the post-acrosomal sheath. This localisation appeared different in patients with infertility: in particular, there was a strong p75^NTR^ signal in the midpiece and the cytoplasmic residue or coiled tails of altered ejaculated sperm. In line with these findings, NGF receptors were intensely expressed in the epididymis and interstitial tissue of the testis. These data suggest the distinctive involvement of NGF and its receptors in the physiology of sperm from fertile men and men with infertility, indicating a possible role for new targeted treatment strategies.

## 1. Introduction

Neurotrophins are a family of growth factors mainly involved in the regulation of neuronal survival, function, and plasticity within the central and peripheral nervous systems. One member, nerve growth factor (NGF), also exerts a variety of effects on non-neuronal cells [1,2,3,4,5,6]. Non-neuronal cells produce this protein, and several cell types throughout the body may express NGF receptors: the high-affinity tropomyosin receptor kinase A (TrKA), responsible for the activation of signalling pathways that promote cell survival, differentiation and growth, and the low-affinity receptor p75 neurotrophin receptor (p75^NTR^), which is more broadly expressed in various cell types where it modulates cellular responses based on the specific context [7,8,9,10].

Researchers have recently hypothesised that NGF is involved in testis morphogenesis and the regulation of spermatogenesis [11,12,13,14]. Moreover, NGF could play a role in the activation of the hypothalamus–pituitary–gonadal axis (HPG) with the secretion of gonadotrophin-releasing hormone (GnRH) [15].

Most of the NGF-related studies on male reproduction have been performed in animals (llamas, alpacas, rabbits, mice, rats, and even adult zebrafish). The results indicate that NGF signalling may play a role in supporting the proliferation and differentiation of spermatogonia and in supporting Sertoli cells [16,17,18,19,20,21,22,23]. In rodents, NGF is involved in the regulation of reproductive behaviours, conditioning sexual conduct and mate choice [24]. In addition, it contributes to the formation and configuration of testicular tissue, mainly during the early stages of testicular development and differentiation of male germ cells [25,26,27,28].

In vitro and in vivo models of male rabbits have shown that NGF exerts its effects on sperm by binding to TrkA and p75^NTR^. TrkA activation by NGF stimulates sperm survival and modulates the acrosome reaction through activation of kinase pathways [20]. On the other hand, the binding of the NGF to p75^NTR^ modulates sperm apoptosis and motility and regulates the sperm respiratory chain [11,19,20,29].

In mice, researchers have shown that NGF influences spermatogenesis through interactions with the nervous system and/or hormonal signalling [30]. NGF and its receptors are widely distributed in the prostate, suggesting potential roles in prostate development, function and pathology [31]. In addition, other authors have shown that the NGF level correlates with the sperm concentration, viability and motility, and the testosterone level [17,20,22,32,33]. Moreover, evidence in men and cattle indicate that the exogenous addition of NGF could be useful for improving sperm quality after cryopreservation [12,34,35,36].

The sperm count in humans has progressively declined in recent decades. Since 1970, there has been about a 50% decrease in the sperm concentration, most notably in Western countries, due to several known and indeterminate reasons [37]. Consistently, human infertility has increased: it affects 8–12% of couples, and the male factor is estimated as a primary or contributing cause in approximately 50% of couples [38].

In this context, recognition of the role NGF plays in the human reproductive system is still in its early stages, and much remains to be described, although the first reports about the presence of NGF and its receptors in the testis are from the 1990s [39].

Li et al. [40] compared fertile men, men with oligoasthenozoospermia, and men with asthenozoospermia and found a significantly lower level (*p* < 0.05) of TrkA messenger RNA (mRNA) in sperm from men with oligoasthenozoospermia compared with sperm from fertile men and men with asthenozoospermia, suggesting a role for NGF in male infertility. It is possible that NGF is associated with specific reproductive pathologies. Indeed, patients with erectile dysfunction and metabolic syndrome show reduced plasma NGF and thiol levels and TrKA expression in white blood cells [41]. In particular, NGF could be involved in reproductive conditions characterised by increased oxidative stress, which generates specific prostaglandin-like products (isoprostanes (IsoPs)) particularly in tissue with a high level of unsaturation in cell membranes (the brain, reproductive tissue and sperm). Seminal F_2_-isoprostanes (F_2_-IsoPs), a class of IsoPs, are considered a marker of sperm immaturity in semen from infertile patients with varicocele [42]. Moreover, interleukin 1β (Il-1β) may be considered a marker of inflammatory reproductive pathologies [43].

In the present study, we evaluated the involvement of NGF in the human male reproductive system by analysing the expression of NGF and both its receptors (TrkA and p75^NTR^) in semen and sperm from fertile men and men with infertility caused by varicocele or urogenital infections (UGIs). We examined the localisation of both NGF receptors in the testis and epididymis to understand whether and how alterations in their presence are associated with specific conditions of male infertility.

## 2. Materials and Methods

### 2.1. Patients

Seminal samples were obtained from 14 men with infertility (aged 31–40 years) attending, from July 2021 to January 2022, the Unit of Medically Assisted Reproduction at Siena University Hospital (Italy) for semen analysis. No pregnancy had ever been declared. The inclusion criteria were normal karyotype, normal follicle-stimulating hormone (FSH), luteinising hormone (LH) and testosterone levels and a body mass index <25 kg/m^2^. The exclusion criteria included azoospermia; a history of cryptorchidism; leucocytospermia; occupational chemical exposure; Y chromosome microdeletions; diabetes; radiotherapy; chemotherapy; use of drugs, alcohol, and dietary supplements; and a heavy smoking habit (>10 cigarettes/day).

The men were categorised according to clinical diagnosis into two groups: a group with varicocele (V group; *n* = 7) or group with UGIs (UGI group; *n* = 7). Scrotal eco-colour Doppler analysis diagnosed the presence of varicocele. Four patients had a left-side grade 2 varicocele, two patients had a left-side grade 3 varicocele, and one patient had a right-side grade 2 varicocele. Patients with a positive bacteriological analysis were included in UGI group. Selected patients showed either varicocele or infections. In the UGI group, two semen samples were positive for *Enterococcus faecalis*, three were positive for *Escherichia coli*, one was positive for *Ureaplasma urealyticum* and one was for *Mycoplasma hominis*. A third group comprised six men (aged 29–40 years) who were fertile donors (F group): they had fathered at least one child during the past 3 years. They had no infections or hormonal and anatomical problems.

All patients and controls provided informed written consent before their inclusion in this study. The study was conducted in accordance with the Declaration of Helsinki, and the protocol was approved by the Ethics Committee of Siena University Hospital (ID CEAVSE 191113).

### 2.2. Semen Analysis

Semen samples were collected by masturbation in a sterile container; samples were examined after liquefaction for 30 min at 37 °C. A part of each semen sample, recovered with sterile pipettes, was sent to the microbiological laboratory (within 1.30 h after collection) as advised in the World Health Organization (WHO) guidelines [44].

The volume, pH, sperm concentration, progressive motility (rapid and slow), normal morphology and viability were assessed as recommended by the WHO [44]. Eosin Y (CI 45380) staining was used to evaluate sperm vitality; more than 300 spermatozoa per sample were examined with a light microscope, recording red-stained cells (dead) and unstained cells (vital). Leukocytospermia was identified with peroxidase staining (>1 × 10^6^ leukocytes/mL).

After the semen evaluation, samples were centrifuged at 400× *g* for 15 min to separate the seminal plasma and the sperm. The aliquots were stored at −80 °C until use.

### 2.3. Aniline Blue (AB) Test

The AB test was performed to evaluate the maturity of the sperm chromatin.

Two hundred microlitres of seminal fluid was washed in phosphate-buffered saline (PBS), centrifuged at 400× *g* for 15 min and resuspended in PBS. The sample, appropriately diluted, was smeared on slides and air-dried. The slides were fixed with 3% glutaraldehyde in PBS (pH 7.2) for 30 min in a humid chamber at room temperature. Then, the slides were treated with AB solution (5% aniline powder and 4% acetic acid in the appropriate volume of distilled water; pH 3.5) for 5 min. After washing in distilled water, the slides were observed under a Leitz Aristoplan microscope (Leica, Wetzlar, Germany). A minimum of 300 spermatozoa per sample were examined.

### 2.4. Gas Chromatography/Negative-Ion Chemical Ionisation Tandem Mass Spectrometry (GC/NICI-MS/MS)

F_2_-IsoPs are initially produced in situ on phospholipids and defined as esterified F_2_-IsoPs. Subsequently, they are released into the circulation as free (non-esterified) F_2_-IsoPs. Total (free plus esterified) F_2_-IsoPs were quantified in seminal plasma with GC/NICI-MS/MS. Butylated hydroxytoluene (BHT) was added (final concentration 90 μM) to each sperm sample at the time of collection. At this stage, the samples were stored at −80 °C until the assay. At the time of F_2_-IsoP detection, basic hydrolysis was performed by incubation (45 °C, 45 min) with 1 N KOH (1:0.5, *v*:*v*). Afterward, the sample was acidified by adding 1 N HCl (1:0.5, *v*:*v*). A tetradeuterated derivative of prostaglandin (PGF_2α_-d4, 500 pg) was added as an internal standard. The sample was purified by using two different solid-phase extractions (octadecylsilane [C18 cartridge] and aminopropyl [NH_2_ cartridge]) to obtain a final eluate to be derivatised before performing GC/NICI-MS/MS. The amount of 8-iso-PGF_2α_, the most abundant isomer for F_2_-IsoP detection (also known as 15-F_2t_-IsoP), was quantified by measuring *m*/*z* 299 production, derived from the [M-181]^−^ precursor ions, and compared with the *m*/*z* 303 ion produced by PGF_2α_-d4 in the applied GC/NICI-MS/MS protocol [45]. For quantification, a calibration curve was constructed using a reference 8-iso-PGF_2α_ compound (Item No. 16350, Caymen Chemical, Ann Arbor, MI, USA).

### 2.5. Enzyme-Linked Immunosorbent Assay (ELISA) 

In seminal plasma, the amount of IL-1β was determined with a sandwich ELISA (Invitrogen, Thermo Fisher Scientific, Waltham, MA, USA). The absorbance at 450 nm was used to determine the IL-1β level in each sample. Curve-fitting software was applied to generate the standard curve (ranging from 0 to 2500 pg/mL) and to quantify IL-1β the samples. All measures were performed in duplicate. The results are expressed as pg/mL.

The NGF concentration was determined with the Human Beta-NGF ELISA Kit (Invitrogen, Thermo Fisher Scientific) according to the manufacturer’s instructions. The standard curve demonstrated a direct relationship between optical density and the NGF concentration. All samples were run in duplicate. The NGF concentration is expressed in pg/mL. The limit of detection was 14 pg/mL.

### 2.6. FACScan Analysis

TrKA and p75^NTR^ were evaluated in semen immediately after collection, as described in a previous paper [46]. Briefly, aliquots of 1 × 10^6^/mL of sperm were placed in tubes and preincubated with PBS/bovine serum albumin (BSA, 0.5%, *w/v*) for 30 min at 4 °C. After washing three times in PBS supplemented with BSA, sperm were incubated at 4 °C for 1 h in PBS/BSA containing FITC-labelled rabbit anti-human p75^NTR^ extracellular domain (ANT-007-F, Alomone Labs, Jerusalem, Israel) (10 mL/sample) and PE-labelled mouse anti-human TrKA (FAB1751P, R&D Systems, Minneapolis, MN, USA) (10 mL/sample). After 1 h, the sperm were washed, resuspended in staining buffer (PBS + 2% foetal bovine serum (FBS) + 1% paraformaldehyde) and analysed by flow cytometry. All procedures were performed at 4 °C. A ‘flame-shaped region’ was established to exclude debris, large cells, and aggregates. Ten thousand live-gated events were collected for each sample, and isotype-matched antibodies were used to determine binding specificity. The results are expressed as the percentage of positive cells/antibodies used for staining (% positive cells). All experiments included a negative control incubated with the Normal Goat IgG Control Mouse IgG Isotype Control (Thermo Fisher Scientific). The analysis was performed with CellQuest Software, version 6.0 (Becton Dickinson, Florence, Italy) and the final figures were prepared with the Kaluza software version 2.1 (Beckman Coulter, Milan, Italy).

### 2.7. Quantitative Polymerase Chain Reaction (qPCR)

Total RNA was extracted from sperm with the RNeasy Mini Kit (Qiagen, Hilden, Germany), based on the manufacturer’s recommendations. Using SuperScript III (Invitrogen, Thermo Fisher Scientific), 1 µg of total RNA was reverse-transcribed into first-strand complementary DNA (cDNA). qPCR was conducted on a StepOne System (Applied Biosystems, Thermo Fisher Scientific) using a PowerUp SYBR Green Real-time PCR Master Mix (A25742 Applied Biosystems, Thermo Fisher Scientific). The primers are shown in Table S1. *GAPDH* was used to normalise gene expression, as described previously [20,47].

### 2.8. Immunofluorescence

p75^NTR^ and TrKA localisation was determined with immunofluorescence. Sperm samples from fertile men and men with infertility were washed in PBS, smeared on glass slides, air-dried, and fixed in 4% paraformaldehyde for 15 min. Two hundred sperm per sample were evaluated.

Tissue sections from adult human testis and epididymis were collected from the archives of the Pathology Department of the University of Siena. Testicular specimens were obtained from the testes of patients who underwent orchiectomy for testicular seminoma before they had received any treatment. The fresh specimens were immediately fixed in 10% buffered formalin for 24 h and submitted to routine processing and paraffin embedding.

The paraffin-embedded sections were deparaffinised with xylene, treated in a graded ethanol series (100%, 90%, 80% and 70%) for 5 min and, finally, incubated in water to rehydrate the tissue. For antigen retrieval, the sections were washed and treated with heat-induced epitope retrieval 1 (HIER 1) buffer (10 mM sodium citrate) at pH 6 for 20 min at 95 °C. After treatment with a blocking solution (PBS containing 1% BSA and 5% normal goat serum (NGS)) for 20 min, the slides were incubated overnight at 4 °C with the following primary monoclonal antibodies diluted 1:100: anti-p75^NTR^ NGF Receptor/CD271 (GeneTex Inc., Irvine, CA, USA) and anti-TrKA (GeneTex Inc.). After three washes in PBS (10 min each), the slides (excluding those treated with conjugated primary antibody) were incubated with goat anti-mouse antibody conjugated to Alexa Fluor 488 (Invitrogen, Thermo Fisher Scientific) for testis and epididymis or goat anti-mouse antibody conjugated to Alexa Fluor 568 (lot. 2124366, Life Technologies Corporation, Eugene, OR, USA) diluted 1:100 for 1 h at room temperature. The slides were washed three times with PBS and then incubated with 4′,6-diamidino-2-phenylindole (DAPI, Sigma-Aldrich, Milan, Italy) for 10 min, followed by washing with PBS for 10 min. Finally, the slides were mounted with 1,4-diazabicyclo [2.2.2]octane (DABCO, Sigma-Aldrich). Observations were made with a Leica DMI 6000 Fluorescence Microscope (Leica Microsystems, Wetzlar, Germany), and images were acquired using the Leica AF6500 Integrated System for Imaging and Analysis (Leica Microsystems). In the testis specimens, the expression of the proteins was examined in the area of histologically non-neoplastic tissue.

### 2.9. Statistical Analysis

The results are expressed as the least squares means and the standard error of the mean (SEM) or the root mean square error (RMSE) [48]. One-way analysis of variance (ANOVA) followed by the Tukey test was used to compare the three groups (F, V and UGI). A *p*-value < 0.05 was considered to indicate a statistically significant difference in the means. Pearson correlation coefficients were determined to assess the correlation between the NGF level and other sperm parameters.

## 3. Results

### 3.1. Main Sperm Traits

The main seminal characteristics of the men, grouped according to their reproductive condition, are shown in Table 1. We evaluated sperm concentration, progressive motility, normal morphology, immaturity, and vitality. Fertile men showed significantly higher (*p* < 0.05) sperm concentration, progressive motility, normal morphology, and vitality compared with men with varicocele or UGIs. Sperm immaturity was significantly increased in men with varicocele compared with fertile men and men with UGIs.

**Figure 1:**
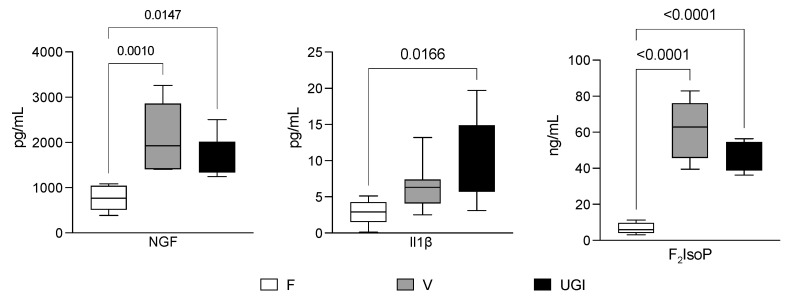
Nerve growth factor (NGF), interleukin 1β (IL-1β) and F_2_-isoprostane (F_2_IsoP) levels in the seminal plasma from fertile men (F) and men with varicocele (V) and urogenital infections (UGIs). The plots display the results obtained by enzyme-linked immunosorbent assays for NGF (image on the left) and IL-1β (image in the middle), and by gas chromatography/negative-ion chemical ionisation tandem mass spectrometry (GC/NICI-MS/MS) for F_2_IsoPs (image on the right). The data were analysed with one-way analysis of variance followed by the Tukey test for multiple comparisons (α = 0.05 and 95% confidence interval). The significant *p* values (<0.05) are indicated.

Figure 1 shows the NGF, IL1β and F_2_IsoP levels in seminal plasma. NGF was significantly higher in men with varicocele or UGI compared with fertile men (2049.0 ± 750.8 pg/mL and 1673.0 ± 457.6 pg/mL vs. 763.3 ± 279.4 pg/mL; *p* = 0.015 and *p* = 0.02, respectively (image on the left). There was not a significant difference in the NGF level between men with varicocele and men with UGIs (*p* = 0.4). Men with UGIs showed a significantly higher IL-1β concentration (10.0 ± 5.6 pg/mL) compared with fertile men (2.8 ± 1.7, *p* = 0.02), but it was not significantly different from men with varicocele (6.6 ± 3.3, *p* = 0.3). Moreover, there was no difference between men with varicocele and fertile men (*p* =0.3) (image in the middle). Finally, men with varicocele had the highest F_2_IsoP level (60.3 ± 15.8 ng/mL). It was significantly higher than the level in fertile men (6.6 ± 3.2 ng/mL, *p* < 0.0001) and men with UGIs (44.5 ± 7.9 ng/mL, *p* = 0.04) (image on the right).

The intra-class correlation coefficients (Table 2) showed a large variation in the different groups, meaning that the association between traits was deeply affected by the reproductive condition (F vs. V vs. UGIs). We noted positive correlations between the NGF level and the F_2_IsoP and IL1β levels in men with varicocele (0.80 and 0.81, respectively, *p* < 0.05) and men with UGIs (0.87 and 0.64, respectively, *p* < 0.05), and between the NGF level and sperm immaturity in all the groups (F, 0.94; V, 0.54; UGIs, 0.78; all *p* < 0.05).

On the contrary, there was a negative correlation between the NGF level and normal sperm morphology in all groups (F, −0.81; V, −0.77; UGIs, −0.85; *p* < 0.05). In men with UGIs, the NGF level correlated negatively with progressive sperm motility (−0.50, *p* < 0.05) and vitality (−0.67, *p* < 0.05). In fertile men, the NGF level correlated positively with progressive motility (0.63, *p* < 0.05).

### 3.2. TrKA and p75^NTR^ Localisation in Sperm from Fertile Men

**Figure 2:**
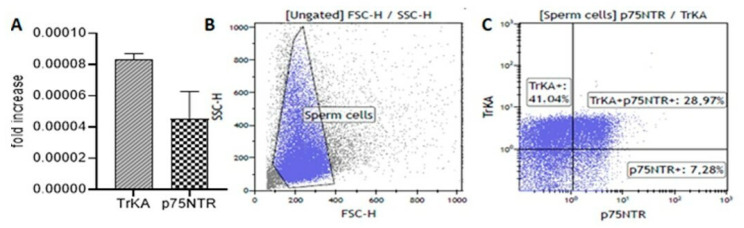

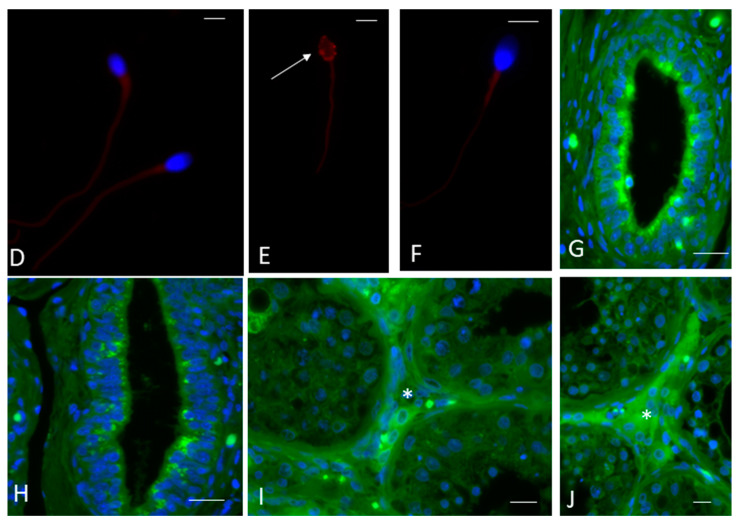
Nerve growth factor (NGF) receptors in the ejaculated sperm, epididymis and testis of fertile men. (**A**) The bar graph shows the mean and standard deviation of the fold increase in TrKA (slanted-line bar graph) and p75^NTR^ (checkerboard bar graph) messenger RNA (mRNA) levels in fertile men (*n* = 7). (**B**,**C**) The plots (blue gate) display the flow cytometric analysis (representative of one fertile man) measuring the size and granularity of sperm (**B**) and their extracellular expression of NGF receptors (**C**). (**D**–**F**) Fluorescence immunostaining of human sperm from fertile men incubated with the anti-TrKA (**D**,**E**) and anti-p75^NTR^ (**F**) antibodies. In (**D**,**E**), the labelling is evident throughout the entire tail and in the post-acrosomal sheath ((**E**), arrow). In (**F**), there is weak staining throughout the entire tail. The nuclei are stained with DAPI, except in (**E**) where the localisation of post-acrosomal sheath is shown. (**G**–**J**) Immunolocalisation of the anti-TrKA and anti-p75^NTR^ antibodies in the epididymis (**G** and **H**, respectively) and testis (**I** and **J**, respectively) from a fertile man. Both receptors appear in an apical position of the epididymal epithelium ((**G**): TrKA, (**H**): p75^NTR^). There is also clear localisation of both antibodies in the interstitial tissue of the testis (asterisks, (**I**): TrKA, (**J**): p75^NTR^). Bars: 5 µm (**D**–**F**), 50 µm (**G**–**J**).

We detected both TrKA and p75^NTR^ in sperm by using several methodological approaches (Figure 2). The TrkA mRNA level in sperm was twice as high as the p75^NTR^ mRNA level (Figure 2A). Furthermore, flow cytometry showed a higher percentage of TrKA-positive sperm (70%) with respect to p75^NTR^-positive sperm (36%) (Figure 2B,C). Again, the TrKA level was consistently higher than the p75^NTR^ level.

Immunofluorescence analysis confirmed the presence and localisation of TrKA and p75^NTR^ receptors in ejaculated sperm. TrKA was localised in the tail and the post-acrosomal sheath of normal sperm (Figure 2D,E). There was weak p75^NTR^ staining throughout the entire tail (Figure 2F). TrKA and p75^NTR^ were also widely present in the epididymis (Figure 2G and 2H, respectively) and the interstitial tissue of the testis (Figure 2I and 2J, respectively).

### 3.3. TrKA and p75^NTR^ Expression in Men with Infertility

**Figure 3:**
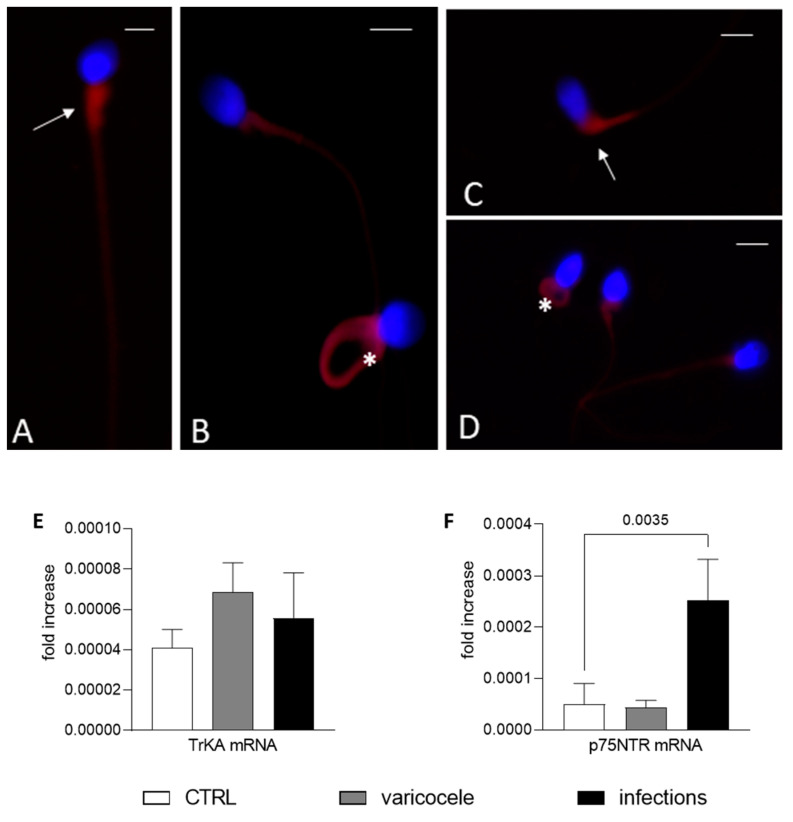
Nerve growth factor (NGF) receptors in altered sperm from men with infertility. (**A**–**D**) Fluorescence immunostaining of human sperm from men with infertility (varicocele (V) or urogenital infections (UGIs)) incubated with the anti-TrKA (**A**,**B**) and anti-p75^NTR^ (**C**,**D**) antibodies. In (**A**) (sperm from the UGI group), the TrkA signal is evident in the midpiece of the sperm (arrow); in (**B**) (sperm from V group), there is staining in the midpiece and in the entire coiled tail (asterisk). In (**C**,**D**), p75^NTR^ staining is strong in the midpiece and cytoplasmic residue (arrow, sperm from the V group) and in the entire coiled tail (asterisk) and midpiece ((**D**), from the UGI group). Bars: 5 µm. (**E**,**F**) The bar graphs show the mean and standard deviation of the fold change in the TrKA (**E**) and p75^NTR^ (**F**) mRNA level for fertile men (*n* = 7, F, white bars), men with varicocele (*n* = 7, V, grey bars) and men with UGI (*n* = 7, black bars). One-way analysis of variance followed by the Tukey test was used for multiple comparisons (α = 0.05 and 95% confidence interval). The significant *p* value (<0.05) is indicated.

In a variable percentage of altered sperm collected from men with varicocele or UGIs, there was strong TrKA staining in the cytoplasmic residue and in the coiled tail (Figure 3A,B). Moreover, there was strong p75^NTR^ staining in altered sperm. In particular, in immature sperm there was staining in round and elliptical heads, cytoplasmic droplets and coiled tails (Figure 3C,D). qPCR showed heterogenous mRNA expression of both NGF receptors. While the TrKA mRNA level was similar in fertile men and men with infertility, the *p75^NTR^* gene was differently expressed in fertile men and men with infertility (Figure 3E,F). Although these differences were significant only for men with UGIs compared with fertile men, these data corroborate the idea that the gene expression of NGF receptors, and in particular *p75^NTR^* (Figure 3F), plays some role in infertility. It should be noted that qPCR detected all the receptors (endogenous and surface).

## 4. Discussion

In this paper, we evaluated the role of NGF and its receptors in the sperm, testis and epididymis of fertile men and men with infertility due to varicocele or a UGI. The main pathological features of varicocele and UGIs are associated with oxidative stress and the inflammatory status of the reproductive apparatus [43,49]. Previous studies in humans and various animal species have analysed the role of seminal NGF on sperm [19,20,29]. Nevertheless, to our knowledge, no other investigation has studied this pattern of qualitative traits (sperm morphology; immaturity; and the profile of NGF receptors in the sperm, testis, and epididymis) in fertile men or men with different reproductive pathologies. NGF can influence sperm characteristics by acting on sperm development during spermatogenesis and on mature cells after ejaculation.

During spermatogenesis, NGF exerts a mitogenic and regulatory effect on Sertoli cells [50] and germ cells [51] in seminiferous tubules [26]. The mechanisms through which NGF affects sperm characteristics have been analysed [52] but not fully clarified. The effect of NGF is mediated by the expression of its receptors (p75^NTR^ and TrKA) in the reproductive tract and in sperm. Both NGF receptors affect testis cord development. Indeed, the p75NTR and TrKA profile of cells play a crucial role in testis morphogenesis, especially during the early stages of development [27,53,54], a phenomenon confirmed by the correlation we found between NGF and immaturity. In particular, the localisation of the two receptors in human testis and epididymis of fertile men confirms the role of NGF during sperm development. Both receptors have been localised in interstitial testicular tissue, indicating the possible interaction of NGF with Leydig cells and endocrine regulation. As described later, reproductive pathologies influence the distribution of NGF receptors and the NGF level in connection with oxidative stress and/or inflammation of the reproductive tissues.

Seminal NGF influences the motility rate, capacitation and acrosome reaction of ejaculated sperm [19,29,32,55]. There is a substantial agreement in the literature regarding the positive effect of NGF (endogenous or exogenous) on the kinetic traits of mature sperm. In Madura bulls, a high seminal NGF level could be considered a good predictor of male fertility after deep freezing [33]; in rabbit bucks, the addition of exogenous NGF (100 ng/mL) improved sperm kinetic traits [19]. Li et al. [40] detected a significantly lower (*p* < 0.05) NGF level in semen from men with oligoasthenozoospermia compared with fertile men and men with asthenozoospermia.

Our findings suggest that the role of seminal NGF cannot be simply considered positive or negative because it is largely affected by the reproductive condition of men (e.g., fertile or infertile with different pathologies). The role of NGF in human sperm appears to be similar to other key molecules such as reactive oxygen species (ROS), F_2-_IsoPs and cytokines. The specific effects depend on the physiological state of cells and the levels of these molecules. In line with these results, our data suggest that in fertile men, a seminal plasma NGF level below a threshold of 1000 pg/mL positively influences spermatogenesis and sperm traits. On the contrary, a NGF level that is too high could be considered a determinant for dysfunction, as shown in men with varicocele and UGIs, who presented an NGF level of 1244 pg/mL and 3260 pg/mL, respectively.

In both pathological conditions, the increase in NGF, which is also involved in inflammation [5,49,56], correlated positively with markers of oxidative damage and inflammation (F_2_IsoPs and IL-1β). Moreover, in men with UGIs, the higher the NGF level was, the lower the number of live and motile sperm. Moretti et al. [57] identified a cut-off for the F_2_-IsoP level in human semen to discriminate among male infertility conditions. In fertile men, we demonstrated a positive correlation (*p* < 0.05) between the NGF level and sperm progressive motility. In these samples, the seminal NGF was also correlated with sperm immaturity and normal morphology. It should be underlined that the level of immaturity and normal morphology in these samples was much better that in the other two groups.

The discrepancy between our results and previous findings could be partly explained by considering the choice of patients [58]. In the present study, we separated the patients based on reproductive pathologies. The simple assessment of the sperm parameter does not seem sufficient to evaluate the role of molecules like NGF on sperm traits. These traits are reduced in almost all the infertility conditions, but the origin of sperm decline (i.e., genetic disease, inflammation or idiopathic infertility) largely affects the role and the influence of the factor studied. Additionally, a comparison with animal experiments should consider that the semen quality in animals is much higher than in humans because the breeding stocks undergo strong selection for reproductive traits. Therefore, bulls, bucks and rams could be compared only with the best semen samples from the fertile group. In this case, the relationship between the NGF level and sperm motility seems much more comparable with previous statements. In accordance with these considerations, we speculated about the effects of NGF in distinct situations (fertile and non-fertile groups with normal or altered sperm traits).

The localisation of both receptors in the tail of mature sperm confirms a role for NGF in sperm motility [11]. TrKA was also localised in the post-acrosomal sheath, which remains integral after the acrosome reaction and is involved in sperm–egg interactions during fertilisation [59,60]. However, a main result is the difference level and localisation of NGF receptors in sperm from fertile men compared with men with infertility. In ejaculated immature sperm, there were strong p75^NTR^ and TrKA signals in the cytoplasmic residue of the head, in the tail and in the mitochondrial region, suggesting a major role for NGF during spermatogenesis. In particular, p75^NTR^ mRNA was highly expressed in infertile sperm samples (mainly from the UGI group) with respect to sperm from fertile men. This finding implies that the normal development of sperm requires a progressive decline in *TrKA* and *p75^NTR^* gene expression. The persistence of a high p75^NTR^ level in mature sperm is a way to reabsorb defective sperm [20].

The high NGF level in men with UGIs exerts a proinflammatory effect by binding to p75^NTR^ rather than TrKA [61]. As expected, NGF is more concentrated during a UGI, consistent with the IL-1β level and inflammatory processes. p75^NTR^ protein and mRNA expression was higher in this group compared with the other two groups. It is largely known that sperm necrosis increases in these patients as well as in semen samples with leucocytospermia [62].

During urogenital infections, the NGF–p75^NTR^ interaction, rather than the NGF–TrKA interaction, modulates pro-apoptotic functions favoured by the high concentrations of inflammatory cytokines and NGF exerting its ‘typical’ growth factor role [5]. This view could contribute to understand why the addition of NGF in sperm dilution media is useful only up to certain concentrations, which generally must not exceed 125 ng/mL [29], and the fact that the effect depends on the ratio of the receptors (pro-apoptotic p75^NTR^ to pro-survival TrkA) [20].

The correlation between the NGF level and motile and live sperm in men with varicocele was less evident; however, this group showed a high NGF level and, as expected, a high level of immature sperm. Varicocele is a sperm pathology associated with inflammation and sperm immaturity [43]. Immature sperm are characterised by uncondensed chromatin [62,63], a coiled tail, the presence of cytoplasmic residue and a high seminal F_2_-IsoP level [58]. We did not measure other markers of oxidation (malondialdehyde, antioxidants and ROS), but the connection between NGF, mitochondrial activity, energy production, ROS and apoptosis has been widely described and theorised [19,29]. Moreover, immature sperm are main sources of ROS [49].

In a rabbit model, we demonstrated that during sperm storage, there is a progressive increase in p75^NTR^ as well as capacitated, necrotic, and apoptotic sperm [20]. All these sperm traits are directly or incidentally connected to ROS production. Sperm movement also enhances cell metabolism and ROS generation [64]. Moreover, capacitation and apoptosis are triggered by high ROS levels [65]. When the concentration of antioxidant molecules and enzymes in seminal plasma or in the cytosol falls and/or the ROS concentration rises, there is increased sperm apoptosis. In turn, apoptosis is mediated by activation of proteins of the BCL-2 family, which recognise the apoptotic stimuli and trigger the permeabilisation of the outer mitochondrial membrane through the BAX and BAK proteins and the release of cytochrome *c* [66]. Furthermore, caspase-9 is activated to form the apoptosome, which initiates the apoptotic cascade [67,68].

From this perspective, we hypothesise that there is also an association between specific reproductive pathologies and the NGF level and the NGF receptor profile in humans, given the increase in sperm apoptosis and necrosis.

Our data suggest that the two NGF receptors act as two different sensors. Although they are able to bind the same molecule, it is possible that upstream, they are sensitive to different concentrations of NGF, and thus, the activation of downstream pathways depends on the NGF concentration.

In this hypothesis, it is likely that physiological NGF levels bind primarily to TrKA and support sperm. Conversely, NGF levels that are too high, connected with some pathological situations, could recruit p75^NTR^ and exert negative effects on sperm traits.

Based on these findings, we can validate the effect of NGF on the quality of human sperm. We propose that the balance between p75^NTR^ and TrKA is a biomarker of good or bad sperm quality. A study with a larger sample size could confirm that the p75^NTR^-to-TrKA ratio is as biomarker of the degree or severity of human pathological conditions such as varicocele, UGIs or others.

We are aware that the number of seminal and testicular specimens should be increased. We must note that we were very selective in choosing the patients; moreover, they had not received any treatment. It should be underlined that in humans, testicular specimens are generally obtained from the testes of patients who have undergone orchiectomy for testicular seminoma, even if the tissue analysed originates from a normal portion of the testis.

## Figures and Tables

**Table 1:** The main sperm traits for the different groups of men.

Group	Sperm Concentration (10^6^/mL)	Sperm Progressive Motility (%)	Sperm Normal Morphology (%)	Sperm Immaturity (%)	Live Sperm (%)
Fertile men	83.6	54.9 a	17.7 b	13.5 a	91.0 a
Men with urogenital infections	57.5	25.8 b	7.3 a	26.2 b	68.6 b
Men with varicocele	64.1	21.4 b	6.2 a	49.0 c	71.8 b
Root mean square error	39.1	11.1	2.4	12.2	5.0

Different letters (a–c) in the same column indicates a significant difference (*p* < 0.05).

**Table 2:** Correlation between the nerve growth factor (NGF) level and other seminal and sperm variables in fertile men (F) and men with varicocele (V) and urogenital infections (UGIs).

Variables		NGF	
	F	UGI	V
Sperm concentration	0.31	−0.72 *	0.55 *
Seminal isoprostanes (F_2_IsoPs)	0.26	0.87 *	0.80 *
Seminal interleukin 1β	0.47	0.64 *	0.81 *
Sperm progressive motility	0.63 *	−0.50 *	0.39
Sperm normal morphology	−0.81 *	−0.85 *	−0.77 *
Sperm immaturity	0.94 *	0.78 *	0.54 *
Live sperm	0.10	−0.67 *	−0.29

* *p* < 0.05.

## Data Availability

The data that support the findings of this study are available from the corresponding author upon reasonable request.

## Supplementary Materials

The following supporting information can be downloaded at: https://www.mdpi.com/article/10.3390/biomedicines11123345/s1, Table S1: list of primers used in the RT-qPCR analysis.
