# Supplementary material for: A Possible Role for Nerve Growth Factor and Its Receptors in Human Sperm Pathology
Source: Biomedicines. 2023 Dec 18;11(12):3345. doi: 10.3390/biomedicines11123345 (PMC10742157; doi:10.3390/biomedicines11123345)
Supplement: Supplementary file 1 [file biomedicines-11-03345-s001.zip › biomedicines-2686514-supplementary.pdf]

Supplementary Table S1. List of primers used in the RT-qPCR analysis

**Transcript**	**Forward Primer 5′-3′**	**Reverse Primer 5′-3′**
TrKA	CAGCCGGCACCGTCTCT	TCCAGGAACTCAGTGAAGATGAAG
$p75^{NTR}$	GAGGCACCTCCAGAACAAGA	GCTGTTCCACCTCTTGAAGG
GAPDH	TCGTGGAAGGACTCATGACC	TCAGCTCAGGGATGACCTTG
